# No relationship between the acromiohumeral distance and pain in adults with subacromial pain syndrome: a systematic review and meta-analysis

**DOI:** 10.1038/s41598-020-76704-z

**Published:** 2020-11-26

**Authors:** Soo Whan Park, Yuan Tai Chen, Lindsay Thompson, Andreas Kjoenoe, Birgit Juul-Kristensen, Vinicius Cavalheri, Leanda McKenna

**Affiliations:** 1 School of Physiotherapy and Exercise Science, Faculty of Health Sciences, Curtin University, Perth, Australia; 2 Research Unit of Musculoskeletal Function and Physiotherapy, Institute of Sports Science and Clinical Biomechanics, University of Southern Denmark, Odense M, Denmark

**Keywords:** Health care, Signs and symptoms

## Abstract

To determine whether subacromial space (i.e. acromiohumeral distance; AHD, and/or occupation ratio percentage) differs between people with subacromial pain syndrome (SAPS) and those without. To investigate whether there is a correlation between subacromial space and pain or disability in adults with SAPS and whether temporal changes in pain or disability are accompanied by changes in subacromial space. Systematic review and meta-analysis. Fifteen studies with a total of 775 participants were included. Twelve studies were of high quality and three studies were of moderate quality using the modified Black and Downs checklist. There was no between group difference in AHD in neutral shoulder position (mean difference [95% CI] 0.28 [−0.13 to 0.69] mm), shoulder abduction at 45° (−0.02 [−0.99 to 0.96] mm) or 60° (−0.20 [−0.61 to 0.20] mm). Compared to the control group, a greater occupation ratio in neutral shoulder position was demonstrated in participants with SAPS (5.14 [1.87 to 8.4] %). There was no consistent pattern regarding the correlation between AHD and pain or disability in participants with SAPS, and no consistent increase in subacromial space with improvement in pain or disability over time. The results suggest that surgical (e.g. sub-acromial decompression) and non-surgical (e.g. manual therapy, taping, stretching and strengthening) management of subacromial pain syndrome should not focus solely on addressing a potential decrease in subacromial space, but also on the importance of other biopsychosocial factors.

## Introduction

Shoulder pain is the third most common musculoskeletal complaint that can lead to disability^1,2^. The prevalence of shoulder pain is reported to range between 7 and 27% in the general population^3^ and even higher in athletic populations involving overhead arm activity, where it can range from 36 to 66%^4–6^.

The most common diagnosis of shoulder pain is subacromial pain syndrome (SAPS)^7^, most often referred to as ‘shoulder impingement syndrome’^8^. The prevalence of SAPS has been suggested to be 36 to 48% of all types of shoulder pain^7,9^. Traditionally, SAPS was thought to have a mechanistic aetiology, where symptoms were caused by ‘impinging’ the subacromial structures against the underside of the acromion through the reduction of the subacromial space. Hence the diagnosis of SAPS was made through the clinical features on physical examination^10^, such as painful arc during shoulder abduction^11^, positive tests of Hawkin’s Kennedy test^12^, Neer’s test^13^, or empty-can test^14^.

However, recently, there has been a debate regarding the mechanism of SAPS as authors in the field have called for a paradigm shift away from using the term ‘impingement’^15,16^ to using “SAPS”^17^ or “rotator cuff-related shoulder pain”^16^. The rationale behind this shift relates to the poor diagnostic accuracy of the individual impingement tests^10^, the complexity of the numerous mechanisms and pathologies associated with pain in the subacromial space^18^, and the negligible reduction in pain with acromiohumeral decompression in comparison to placebo^19^. If impingement between acromion and humerus is not a mechanism for the development of SAPS, then subacromial space should not be related to symptoms, and this needs to be systematically explored.

Subacromial space has been quantified by acromiohumeral distance (AHD) and occupation ratio percentage. Acromiohumeral distance is defined as the shortest distance between the surface of the proximal humerus and the underside of the acromion^20^. Occupation ratio percentage is calculated by dividing the supraspinatus tendon thickness by AHD (i.e. (supraspinatus tendon thickness/AHD) × 100)^21^. Ultrasonography (US), X-ray and magnetic resonance imaging (MRI) techniques have shown to have good reliability and concurrent validity as tools to measure subacromial space^22–24^. Some authors have found no significant difference in AHD between adults with SAPS compared to controls with no shoulder pain^25–30^ whilst others have shown otherwise^31,32^. Thus there is a need for data pooling to provide a better indication of the relationship.

A recently published systematic review^33^ found conflicting results regarding the association between imaging findings of the shoulder such as AHD and symptoms. This study only presented the results narratively, with no meta-analysis investigating the subacromial space differences between adults with SAPS and controls with no shoulder pain. Moreover, they only found three studies and were not able to subgroup data for athletes, which is important, as this group has a higher incidence of shoulder pain^3–6^ and there is a need to better understand mechanistic relationships in this high risk group. Thus, a meta-analysis that additionally includes an examination of athletic populations remains necessary.

In line with the traditional mechanistic theory, adults with SAPS have been managed with the primary goal of increasing AHD. Surgical interventions may often include subacromial decompression surgery^19,24^, while non-surgical management may include a variety of interventions, all with the aim to increase AHD, which could include e.g. manual therapy^29^, kinesio-taping^34^, strengthening^29^, and stretching exercises^35^. However, despite clinical evidence showing some of these interventions to be effective in reducing pain or disability in adults with SAPS, the potential associated mechanisms in subacromial space remain unclear. Furthermore, there is limited knowledge regarding the clinical significance of subacromial space in athletes with SAPS. Therefore, it seems beneficial to summarise the research regarding the cross-sectional and temporal relationship between subacromial space and pain or disability, in order to help guide clinicians to decide the best management for SAPS in both the athletic and general population.

Therefore, we have developed three aims to synthesise the literature regarding subacromial space in adults with SAPS. The first aim of this systematic review is to investigate whether subacromial space differs between adults (including athletes) with SAPS compared to asymptomatic controls. The second aim is to determine if there is a linear correlation between AHD and pain or disability in adults with SAPS. The third aim is to investigate if changes in AHD over time, are accompanied by changes in pain or disability.

## Methods

### Search strategy

This review has been registered in the PROSPERO database (CRD42018103100) and conducted according to the Preferred Reporting Items for Systematic reviews and Meta-Analyses (PRISMA) guidelines^36^. Electronic searches were conducted through the following databases: Cochrane Central Register of Controlled Trials (CENTRAL), PEDro, PubMed and Embase (via Ovid). A systematic search was conducted on the 17^th^ of June 2019. The full search strategy, which was developed in consultation with an information specialist, is outlined in the Appendix. It was adapted for use with each database.

The reference lists of the included studies were reviewed for additional articles not found in the database search. More recent research that has yet to be published was identified through a hand search of abstracts presented between January 2016 and December 2019, in conferences of the American College of Sports Medicine (ACSM), Australasian Musculoskeletal Imaging Group (AMSIG), European Federation of National Associations of Orthopaedics and Traumatology (EFORT), International Symposium on Current Concepts in Knee and Shoulder Arthroscopic Surgery and Arthroplasty (ISKSAA) and the American Orthopaedic Association (AOA). These conferences were identified as most relevant to this topic.

### Types of studies, participants and outcomes

For all three aims, the same participant and outcome measure eligibility criteria were used. Studies were included irrespective of publication type. The studies were eligible for inclusion if the participants had been clinically diagnosed with SAPS and were more than 18 years of age. The clinical diagnosis of SAPS was defined as pain in the shoulder region with any of the following; painful arc^11^ during shoulder abduction, positive Hawkin’s Kennedy test^12^, Neer’s test^13^, or empty-can test^14^. The eligible studies had to include a measurement of subacromial space, such as AHD or occupation ratio that could be measured by MRI, X-ray or US. These measurements had to be taken in upright positions of standing or sitting with the shoulder in neutral position or abduction. The studies were excluded if the shoulder was rotated, e.g. measured in hands behind back or empty-can position^14^. Studies that included participants with pain due to adhesive capsulitis, trauma, rheumatological or neurological conditions were excluded.

The types of studies that were eligible for the first aim were those that included a group with SAPS and a group without SAPS, such as controlled trials, longitudinal, case-cohort and case–control studies. Studies had to include a measure of subacromial space in both participants with clinically diagnosed SAPS and those with no shoulder pain. Disability was not investigated in this aim, as the focus was to observe the relationship between the subacromial space and pain.

The types of studies that were eligible for the second aim included cross-sectional studies that reported a correlation analysis between AHD and self-reported pain or disability in adults with clinical diagnosis of SAPS. Studies were eligible for inclusion for this aim if they reported the use of a patient reported functional outcome measure with established psychometric properties, such as the Constant score^37^, Disabilities of the Arm, Shoulder and Hand (DASH)^38^, Shoulder Pain and Disability Index (SPADI)^39^ or Western Ontario Rotator Cuff (WORC)^40^ questionnaire. The Constant score is a measure of shoulder function by assessing self-reported pain, and activities of daily living within the past week, besides range of motion and strength. It is scored between 0 and 100 where higher scores indicate better outcome. The DASH questionnaire measures self-reported function during the past week and is measured between 0 and 100 where higher scores indicate poorer function. The SPADI questionnaire measures current pain and disability and it is measured as a percentage of 100 where higher scores indicate higher pain and disability. WORC index assesses self-reported physical symptoms, function, emotion and lifestyle during the past week. The final WORC score is converted to a percentage out of 100 where higher percentages indicate better outcome. Other outcome measures for pain such as Visual Analogue Scale (VAS) where higher scores indicate poorer outcome were also eligible for inclusion.

The types of studies that were eligible for the third aim were randomised and non-randomised controlled trials, pre-post designs, prospective and case–control studies that provided baseline and follow-up data. Measures of subacromial space and self-reported pain or disability had to be measured at the same time point in adults with clinically diagnosed SAPS. As for second aim, studies that used recognised functional scales were eligible.

### Study selection

The articles identified from the database and hand search were imported into EndNote^41^ and duplicates were removed. Subsequently, the articles were imported into Covidence^42^. Two review authors used Covidence to independently screen titles and abstracts of all studies identified through the search to determine their eligibility. Upon agreement the remaining articles were full text reviewed by two review authors, and a decision was made for inclusion or exclusion. Any disagreements between the two review authors during screening by title and abstract as well as screening by full text were resolved through a third review author.

### Quality assessment

The studies included in this systematic review were assessed for methodological quality using the modified Downs and Black checklist^43^. Two checklists were identified by the Cochrane Handbook^44^ for the appraisal of randomised and non-randomised studies. The Downs and Black checklist has been shown to be valid and reliable, with satisfactory intra-rater reliability (*r* = 0.88) and inter-rater reliability (*r* = 0.75)^45^. The other recommended checklist, Newcastle–Ottawa scale, has been questioned for its validity^46^, and therefore Downs and Black checklist was chosen.

The modified Downs and Black checklist contains 27 yes/no questions appraising five sections; reporting, external validity, internal validity, confounding (selection bias) and power of the study. Item 27 (power of the study) of the modified Downs and Black checklist^47^, has been altered to have a maximum score of one, and item 5 (distribution of principal confounders) has a maximum score of two. The remaining items are scored as follows: no = 0, yes = 1.

This systematic review included studies with a broad range of study designs and not all questions from the Downs and Black checklist were applicable for all the study designs. Therefore, the total score of the Downs and Black checklist varied according to the respective study design. The maximum possible scores were 28 for RCTs, 26 for case–control and 16 for observational studies. The results from this quality assessment were reported and displayed as percentages of the total modified score. The quality of the studies was rated as follows: low (≤ 33.3%), moderate (33.4–66.7%) or high (≥ 66.8%)^48^. This rating method is consistent with previous systematic review methods^49^.

### Data extraction

Three authors independently extracted data from the included studies using a standardised form. The extracted data was checked by a fourth review author. The number of participants and participant characteristics were extracted for all the studies. The method used to measure the subacromial space was also extracted for all studies.

To address the first aim, all measurements of AHD, occupation ratio and supraspinatus tendon thickness were extracted for both groups from the included studies. With regards to the second aim, the correlation coefficients representing the associations between AHD and pain or disability, were extracted. For the third aim, AHD and pain or disability measurements at both baseline and follow up time points for the experimental group were extracted. The authors of the selected studies were contacted for any missing data of the measurements described.

### Data synthesis

Meta-analyses were performed to address the first aim. Mean ± standard deviations (SD) of subacromial space (at neutral and shoulder abduction) were included in the analyses. Review Manager (RevMan) version 5.3.5^50^ was used for generating the forest plots and for statistical analysis. A fixed effects model was used if no substantial heterogeneity (defined below) was detected, otherwise a random effects model was used. The meta-analyses used the inverse variance Der Simonian and Laird method^51^. Mean differences (MD) with 95% confidence interval (CI) were calculated. A subgroup analysis was further performed for the athletic population to address the first aim. Results of the studies that addressed the second and third aims were presented as a narrative synthesis. Where statistical significance was not reported in studies that addressed the third aim, an effect size calculator (https://www.cem.org/effect-size-calculator) was used to calculate significance.

### Assessment of heterogeneity and sensitivity analysis

Heterogeneity across studies that addressed the first aim was examined by visual analysis of the forest plots and the I^2^ test produced through RevMan^50^. An I^2^ > 50% denoted substantial heterogeneity^44^. Where substantial heterogeneity was detected, a sensitivity analysis was undertaken to investigate methodological differences across studies.

## Results

**Figure 1:**
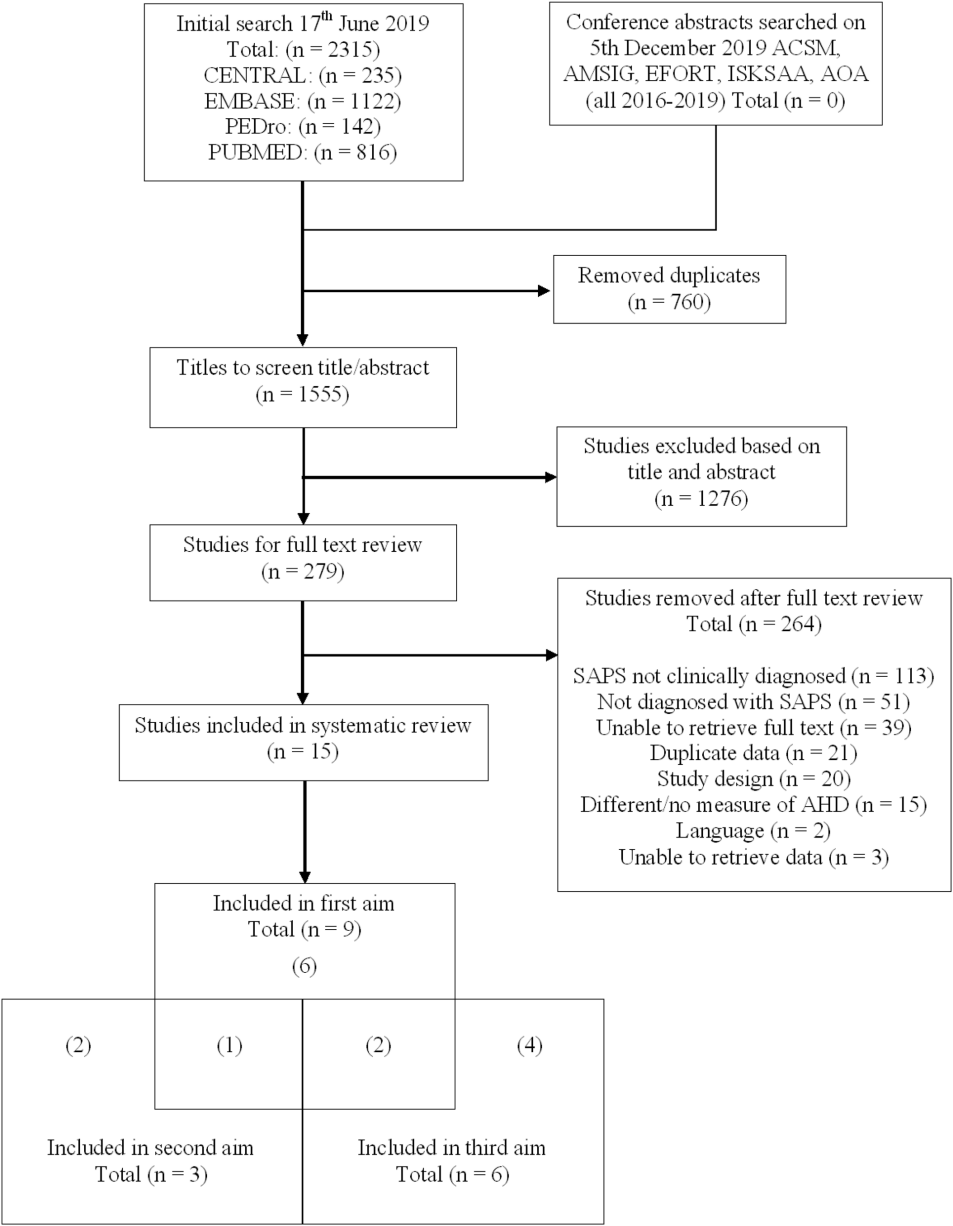
Flow chart of included studies. From the nine studies included in the first aim, one study was also included in the second aim. Similarly, two studies were also included for the third aim.

The search identified 2315 records (Fig. 1). After removing duplicates and screening records by title and abstract, the full texts of the remaining 279 records were reviewed. A total of 264 studies were excluded during full text review. The most common reason for exclusion was that the participants were diagnosed with SAPS using imaging rather than clinical tests. Another common reason for exclusion was that the participants were not diagnosed with SAPS. Fifteen studies met the inclusion criteria; nine of the 15 addressed the first aim^21,29,31,32,52–56^, three addressed the second aim^54,57,58^ and six addressed the final aim^29,32,59–62^. Of the nine studies that addressed the first aim, one also addressed the second aim^54^ and two addressed the third aim^29,32^.

### Study characteristics

A total of 775 participants were included across the 15 studies (Tables 1, 2, and 3). In the nine studies that addressed the first aim^21,29,31,32,52–56^, 472 participants were included (mean age (SD) ranging between 23 (4) and 65 (10) years). Three of nine studies (170 participants) only included athletes, drawn from a variety of sports^54–56^, and the remaining studies included the general population. The sports included archery, climbing, fencing, badminton, rugby, handball, tennis, water polo and swimming. Eight studies (652 participants) measured AHD and supraspinatus tendon thickness by US^21,29,32,52–56^, whilst one study measured AHD through X-ray^31^. Three of the studies (64 participants) that measured supraspinatus tendon thickness by US also calculated the occupation ratio percentage^21,52,55^.

**Table 1:** Characteristics of included studies in the first aim.

Author Study design	Method of measuring AHD	Population type	Population characteristics (Age: Mean ± SD years unless denoted)	Results (mean ± SD, mm)
**First aim**				
Benitez-Martinez et al.^54^ Cross-sectional	US	Athletes	Pain group: n = 38 (38 males) Age: 28.26 ± 8.4 Pain duration: not reported No pain group: n = 43 (43 males) Age: 24.21 ± 7.9	AHD in 0° shoulder abduction: Pain group: 9.10 ± 1.30 No pain group: 9.10 ± 1.30
de Witte et al.^31^ Case–control	X-Ray	General	Pain group: n = 28 (11 males) Age: 50.1 ± 1.6 Pain duration: > 3 months No pain group: n = 10 (5 males) Age: 50.2 ± 6.6	AHD in 0° shoulder abduction: Pain group: 11.10 ± 1.84 No pain group: 8.90 ± 1.92
Desmeules et al.^32^ Case–control	US	General	Pain group: n = 7 (gender not reported) Age: 44 ± 3.8 Pain duration: 4–24 weeks No pain group: n = 13 (gender not reported) Age: 34 ± 9	AHD in 0° shoulder abduction: Pain group:12.00 ± 1.90 No pain group: 9.90 ± 1.50 AHD in 45° shoulder abduction: Pain group: 9.50 ± 2.70 No pain group: 8.30 ± 1.90 AHD in 60° shoulder abduction: Pain group: 9.60 ± 2.30 No pain group: 7.60 ± 1.70
Hougs Kjaer et al.^55^ Case–control	US	Athletes	Pain group: n = 22 (12 males) Age: 38 (range 18–56) Pain duration: Not reported No pain group: n = 24 (9 males) Age: 36 (range 19–57)	AHD in 0° shoulder abduction: Pain group:10.56 ± 1.36 No pain group: 10.89 ± 1.34 STT (mm) in 0° shoulder abduction: Pain group: 5.82 ± 1.28^†^ No pain group: 5.54 ± 0.57^†^ OcR (%) in 0° shoulder abduction: Pain group: 55.19 ± 10.51%^†^ No pain group: 51.61 ± 8.08%^†^
Leong et al.^56^ Case–control crossover trial	US	Athletes	Pain group: n = 26 (26 males) Age: 23.6 ± 3.3 Pain duration: > 3 months No pain group: n = 17 (17 males) Age: 21.7 ± 3.5	AHD in 0° shoulder abduction: Pain group: 8.70 ± 1.10 No pain group: 8.30 ± 0.80 AHD in 60° shoulder abduction: Pain group: 5.80 ± 1.70 No pain group: 6.20 ± 1.40
McCreesh et al.^52^ Case–control	US	General	Pain group: n = 23 (12 males) Age: 47 ± 12.8 Pain duration: 13 ± 8.4 months No pain group: n = 20 (10 males) Age: 43 ± 9.5	AHD in 0° shoulder abduction: Pain group: 10.04 ± 1.06 No pain group: 9.58 ± 1.19 STT (mm) in 0° shoulder abduction: Pain group: 5.60 ± 0.88 No pain group: 4.96 ± 0.67 OcR (%) in 0° shoulder abduction: Pain group: 56.61 ± 11.66%^†^ No pain group: 52.22 ± 7.66%^†^
Michener et al.^21^ Single-blind cross-sectional	US	General	Pain group: n = 20 (10 males) Age: 45.1 ± 11.1 Pain duration: > 6 weeks No pain group: n = 20 (10 males) Age: 45.0 ± 11.1	AHD in 0° shoulder abduction: Pain group: 10.80 ± 1.60 No pain group: 11.40 ± 1.70 STT (mm) in 0° shoulder abduction: Pain group: 6.60 ± 0.80 No pain group: 6.00 ± 0.80 OcR (%) in 0° shoulder abduction: Pain group: 61.7 ± 10.3% No pain group: 54.2 ± 7.9%
Navarro-Ledesma et al.^53^ Cross-sectional	US	General	Pain group: n = 76 (26 males) Age: 45.7 ± 10.3 Pain duration: > 3 months No pain group n = 40 (21 males) Age: 46.4 ± 7.0	AHD in 0° shoulder abduction: Pain group: 9.46 (95% CI 9.12–9.79) No pain group: 9.52 (95% CI 9.15–9.89) AHD in 60° shoulder abduction: Pain group: 6.38 (95% CI 6.01–6.75) No pain group: 6.71 (95% CI 6.33–7.09)
Savoie et al.^29^ Prospective pre-post with control group	US	General	Pain group: n = 25 (15 males) Age: 42.6 ± 11.5 Pain duration: 99.3 ± 141.3 weeks No pain group n = 20 (11 males) Age: 39.2 ± 9.5	AHD in 0° shoulder abduction: Pain group: 9.90 ± 2.00 mm No pain group: 9.70 ± 1.50 mm AHD in 45° shoulder abduction: Pain group: 7.90 ± 2.20 mm No pain group: 8.20 ± 1.50 mm AHD in 60° shoulder abduction: Pain group: 7.50 ± 2.10 mm No pain group: 7.70 ± 1.50 mm

^†^Data provided by the author; n: number of participants; AHD: acromiohumeral distance (mm); CI: confidence interval; MRI: magnetic resonance imaging; OcR: Occupation ratio percentage; STT: supraspinatus tendon thickness; US: ultrasound.

**Table 2:** Characteristics of included studies in the second aim.

Author Study design	Method of measuring AHD	Population Type	Population characteristics (Age: Mean ± SD years unless denoted)	Results
**Second aim**				
Benitez-Martinez et al.^54^ Cross-sectional	US	Athletes	n = 38 (38 males) Age: 28.26 ± 8.4 Pain duration: not reported	Correlation between AHD and current pain (VAS): *r* = 0.11, *p* = 0.33
Mayerhoefer et al.^57^ Cross-sectional	MRI + X-Ray	General	n = 47 (33 males) Age: 51.7(range 29–74) Pain duration: > 6 months	Correlation between AHD and Constant score: When AHD measured on X-ray: *r* = 0.39, p = 0.006* When AHD measured on MRI: *r* = 0.41, p = 0.004*
Navarro-Ledesma et al.^58^ Cross-sectional	US	General	n = 97 (34 males) Age: 45.4 ± 8.9 Pain duration: > 3 months	Correlation between AHD and SPADI: In 0° shoulder abduction: *r* = −0.222, p < 0.05* In 60°shoulder abduction: *r* = −0.115, *p* > 0.05

*Indicates significant correlation (*p* < 0.05). ^†^Data provided by the author.

n: number of participants; AHD: acromiohumeral distance (mm); MRI: magnetic resonance imaging; SPADI: Shoulder Pain and Disability Index; US: ultrasound; VAS: Visual Analogue Scale.

**Table 3:** Characteristics of included studies in the third aim.

Author Study design	Method of measuring AHD and types of interventions	Population type	Population characteristics (Age: Mean ± SD years unless denoted)	Results (Mean ± SD mm)	
**Third aim**					
Akkaya et al.^59^ Randomised control and single blinded study	US Experimental group: Weighted pendulum exercise Control group: Unweighted exercises (4 weeks, 3 times daily)	General	Experimental group: n = 18 (6 males) Age: 42.9 ± 8.5 Pain duration: 7.2 ± 4.3 months Control group: n = 16 (5 males) Age: 40.4 ± 9.4 Pain duration: 6.6 ± 4.1 months	Experimental group: AHD in 0° shoulder abduction: Pre:11.20 ± 2.30 Post: 11.10 ± 2.00 *p* = 0.887 AHD in 30° shoulder abduction: Pre: 10.50 ± 1.90 Post: 10.60 ± 1.90 *p* = 0.257 AHD in 60° shoulder abduction: Pre: 10.30 ± 1.90 Post: 10.50 ± 2.20 *p* = 0.571	Experimental group: SPADI: Pre: 62.0 ± 21.5 Post: 32.4 ± 18.7 p = 0.001* VAS rest: Pre: 2.4 ± 2.1 Post: 0.9 ± 1.2 p = 0.006*
Belley et al.^60^ Triple-blind randomised control trial	US Experimental group: Rehab program focused on sensorimotor training with a-tDCS treatment Control group: Rehab program focused on sensorimotor training with sham a-tDCS treatment (6 weeks, 8 treatments with home exercises	General	Experimental group: n = 20 (11 males) Age: 44 ± 11.0 Pain duration: Not reported Control group: n = 20 (11 males) Age: 47 ± 9.0 Pain duration: Not reported	Experimental group: AHD in 0° shoulder abduction: Pre: 10.80 ± 1.70 Post: 11.00 ± 1.40 *p* > 0.05 AHD in 45° shoulder abduction: Pre: 8.90 ± 1.40 Post: 9.50 ± 1.50 p < 0.05* AHD in 60° shoulder abduction: Pre: 8.60 ± 1.40 Post: 9.30 ± 1.50 p < 0.05*	Experimental group: DASH: Pre: 33.0 ± 13.6 Post: 9.5 ± 9.6 p < 0.05* WORC(%): Pre: 53.8 ± 18.1 Post: 89.7 ± 12.5 p < 0.05*
Boudreau et al.^61^ single blinded prospective RCT	US Experimental: EMG-based co-activation of serratus anterior, trapezius, pectoralis major and latissimus dorsi muscles during rotator cuff strengthening program Control: EMG-based rotator cuff strengthening program with no co-activation of serratus anterior, trapezius, pectoralis major and latissimus dorsi (6 weeks, 7 days/wk)	General	Experimental group: n = 21 (12 males) Age: 50.2 ± 10.9 Pain duration: 44.2 ± 52.9 months Control group: n = 21 (8 males) Age: 49.6 ± 13.2 Pain duration: 41.8 ± 40.5 months	Experimental group AHD in 0° shoulder abduction: Pre: 10.8 ± 2.1 Post: 11.5 ± 2.7 *p* = 0*.*56 AHD in 30° shoulder abduction: Pre: 10.4 ± 2.3 Post: 10.6 ± 2.4 *p* = 0*.*44 AHD in 60° shoulder abduction: Pre: 9.5 ± 2.7 Post: 9.8 ± 2.4 *p* = 0*.*75	Experimental group: DASH: Pre: 32.2 ± 15.4 Post: 27.8 ± 18.7 *p* > 0.211 WORC(%): Pre: 51.6 ± 18.5 Post: 65.7 ± 25.8 p < 0.001* VAS movement: Pre: 71.1 ± 17.3 Post: 56.1 ± 29.6 p < 0.001*
Desmeules et al.^32^ Pre-post treatment clinical trial, single group design	US Experimental group: Rehabilitation program involving education, ice, stretching, elastic band exercise for the rotator cuff, postural exercise, Maitland mobilisations, ST and GH control exercises Control group: None (4 weeks, 12 sessions)	General	Experimental group: n = 7 (gender not reported) Age: 44 ± 3.8 Pain duration: 4–24 weeks	Correlation between the maximum change in AHD from shoulder abduction 0–60° with change in WORC%: *r* = 0.84 p = 0.01*	
Dupuis et al.^62^ Parallel group RCT	US Experimental group: Isometric rotator cuff exercises cryotherapy Control group: rest, ice, advice to avoid pain (2 weeks)	General	Experimental group: n = 20 (13 males) Age: 33 ± 7 Pain duration: 27 ± 9 days Control group: n = 23 (11 males) Age: 43 ± 13 Pain duration: 25 ± 7 days	Experimental group: AHD in 0° shoulder abduction: Pre:10 ± 2 Post: 10 ± 1 *p* > 0.11 AHD in 45° shoulder abduction: Pre: 8 ± 2 Post: 8 ± 2 *p* > 0.11 AHD in 60° shoulder abduction: Pre: 7 ± 2 Post: 7 ± 2 *p* > 0.11	Experimental group: DASH: Pre: 29.3 ± 12.6 Post: 16.3 ± 11.3 ***p < 0.05*** WORC(%): Pre: 55.7 ± 21.0 Post: 80.6 ± 17.1 ***p < 0.05***
Savoie et al.^29^ Single group prospective	US Experimental group: Rehabilitation program involving movement training, manual therapy, strengthening and stretching exercises, and patient education Control group: None (6 weeks)	General	Experimental group: n = 25 (15 males) Age: 42.6 ± 11.5 Pain duration: 99.3 ± 141.3 weeks	Experimental group: AHD in 0° shoulder abduction: Pre: 9.9 ± 2.0 Post: 10.4 ± 1.7 *p* > 0.05 AHD in 45° shoulder abduction: Pre: 7.9 ± 2.2 Post: 8.8 ± 1.6 p < 0.05* AHD in 60° shoulder abduction: Pre: 7.5 ± 2.1 Post: 8.2 ± 1.7 p < 0.05*	Change in pain or disability in the experimental group: DASH: 17.1 ± 12.4, p < 0.001* WORC(%): -30.1 ± 14.0, p < 0.001*

*Indicates significant improvement (*p* < 0.05). ^†^Data provided by the author.

N: number of participants; AHD: acromiohumeral distance (mm); a-tDCS: anodal transcranial direct current stimulation; CI: confidence interval; DASH: Disabilities of the Arm Shoulder and Hand questionnaire; GH: gleno-humeral; MRI: magnetic resonance imaging; NA: not applicable; OcR: Occupation ratio percentage; SAPS: subacromial pain syndrome; SIS: subacromial impingement syndrome; SPADI: Shoulder Pain and Disability Index; ST: scapulo-thoracic; STT: supraspinatus tendon thickness; RC: rotator cuff; RCRSP: rotator cuff related shoulder pain; RCT: rotator cuff tendinopathy; US: ultrasound; VAS: Visual Analogue Scale; WORC: Western Ontario Rotator Cuff index.

In the three studies that addressed the second aim^54,57,58^, 182 participants were included (mean age (SD) ranging between 26 (8) to 52 (no SD reported) years old). One study included an athletic population from overhead sports^54^, and the remaining two studies were derived from the general population^57,58^. Two of three included studies measured AHD by US^54,58^ while one study measured AHD through X-ray and MRI^61^.

In the six studies that addressed the third aim^29,32,59–62^, 224 participants were included (mean age (SD) ranging from 39 (10) to 50 (11) years). All 224 participants reported changes in pain or disability along with measurements of subacromial space. All of these included studies recruited participants from the general population and they were tested at follow-up after two to six weeks. All of these studies measured AHD through US.

### Risk of bias

In general, the quality of the studies ranged between moderate and high (Table 4). Twelve studies were of high quality^21,31,53–62^ and three studies were of moderate quality^29,32,52^. All studies, except for one^60^, scored poorly for external validity; items 11–13. These studies did not report the proportion of the source population from which the patients were derived and proportion of the sample that was included. Six studies adjusted their analyses for confounding factors^21,54–56,59,61^, and six studies performed a sample size calculation^21,53,56,60–62^.

**Table 4:** Critical appraisal of the included articles using a modified Downs and Black checklist^43^.

Author	Q 1	Q2	Q3	Q4	Q5*	Q6	Q7	Q8	Q9	Q10	Q11	Q12	Q13	Q14	Q15	Q16	Q17	Q18	Q19	Q20	Q21	Q22	Q23	Q24	Q25	Q26	Q27	Score	%	Quality
Akkaya et al.^59^	Y	Y	Y	Y	Y	Y	Y	Y	Y	Y	N	N	Y	N	Y	Y	Y	Y	Y	Y	Y	N	Y	N	Y	Y	N	22/28	78.5	High
Belley et al.^60^	Y	Y	Y	Y	Y	Y	Y	Y	Y	N	Y	Y	Y	Y	Y	Y	Y	Y	Y	Y	Y	Y	Y	Y	N	Y	Y	26/28	93	High
Benitez-Martinez et al.^54^	Y	Y	Y	X	Y	Y	Y	X	X	Y	N	N	X	X	Y	Y	X	Y	X	Y	X	X	X	X	Y	X	N	13/16	81.5	High
Boudreau et al.^61^	Y	Y	Y	Y	Y	Y	N	Y	N	Y	N	N	N	Y	Y	Y	Y	Y	Y	Y	Y	Y	Y	Y	N	Y	Y	22/28	78.6	High
de Witte et al.^31^	Y	Y	Y	X	Y	Y	Y	X	X	Y	N	Y	X	X	N	Y	X	Y	X	Y	X	X	X	X	N	X	N	12/16	75	High
Desmeules et al.^32^	Y	Y	N	Y	N	Y	Y	N	N	Y	N	N	Y	N	Y	Y	Y	Y	Y	Y	N	Y	X	X	N	N	N	14/26	54	Moderate
Dupuis et al.^62^	Y	Y	Y	Y	Y	Y	Y	Y	Y	Y	N	N	N	N	Y	Y	N	Y	Y	Y	Y	Y	Y	Y	N	Y	Y	22/28	78.6	High
Hougs Kjaer et al.^55^	Y	Y	Y	X	Y	Y	Y	X	X	Y	N	Y	X	X	Y	Y	X	Y	X	Y	X	X	X	X	Y	X	N	14/16	87.5	High
Leong et al.^56^	Y	Y	Y	Y	Y	Y	Y	N	Y	Y	N	N	N	N	Y	Y	Y	Y	Y	Y	Y	N	Y	N	Y	Y	Y	21/28	75	High
Mayerhoefer et al.^57^	Y	Y	Y	X	N	Y	Y	X	X	Y	N	Y	X	X	Y	Y	X	Y	X	Y	X	X	X	X	N	X	N	11/16	69	High
McCreesh et al.^52^	Y	Y	Y	Y	Y	Y	Y	N	N	Y	N	N	N	N	Y	Y	N	Y	Y	Y	Y	N	X	X	N	N	N	15/26	58	Moderate
Michener et al.^21^	Y	Y	Y	X	Y	Y	Y	X	X	Y	N	N	X	X	Y	Y	X	Y	X	Y	X	X	X	X	Y	X	Y	14/16	87.5	High
Navarro-Ledesma et al.^58^	Y	Y	Y	X	Y	Y	Y	X	X	Y	N	N	X	X	Y	Y	X	Y	X	Y	X	X	X	X	N	X	N	12/16	75	High
Navarro-Ledesma et al.^53^	Y	Y	Y	X	Y	Y	Y	X	X	Y	N	N	X	X	Y	Y	X	Y	X	Y	X	X	X	X	N	X	Y	13/16	81.5	High
Savoie et al.^29^	Y	Y	Y	X	Y	Y	Y	Y	Y	N	N	N	Y	N	Y	Y	Y	Y	Y	Y	N	N	X	X	N	Y	N	17/26	65.5	Moderate

Q = Question, N = no or “unable to determine” (score = 0), X = not applicable (score = 0) and Y = yes (score = 1). The total score varies between the studies, where RCT (= 28), case–control (= 26) or observational study design (= 16) give different scores. Quality is evaluated as low (≤ 33.3%), moderate (33.4–66.7%) or high (≥ 66.8%).

*Question 5 includes no (= 0), partially (= 1) or yes (= 2) and therefore potentially scores a maximum of 2 points.

### First aim: Differences in subacromial space between adults with SAPS and those without SAPS

**Figure 2:**
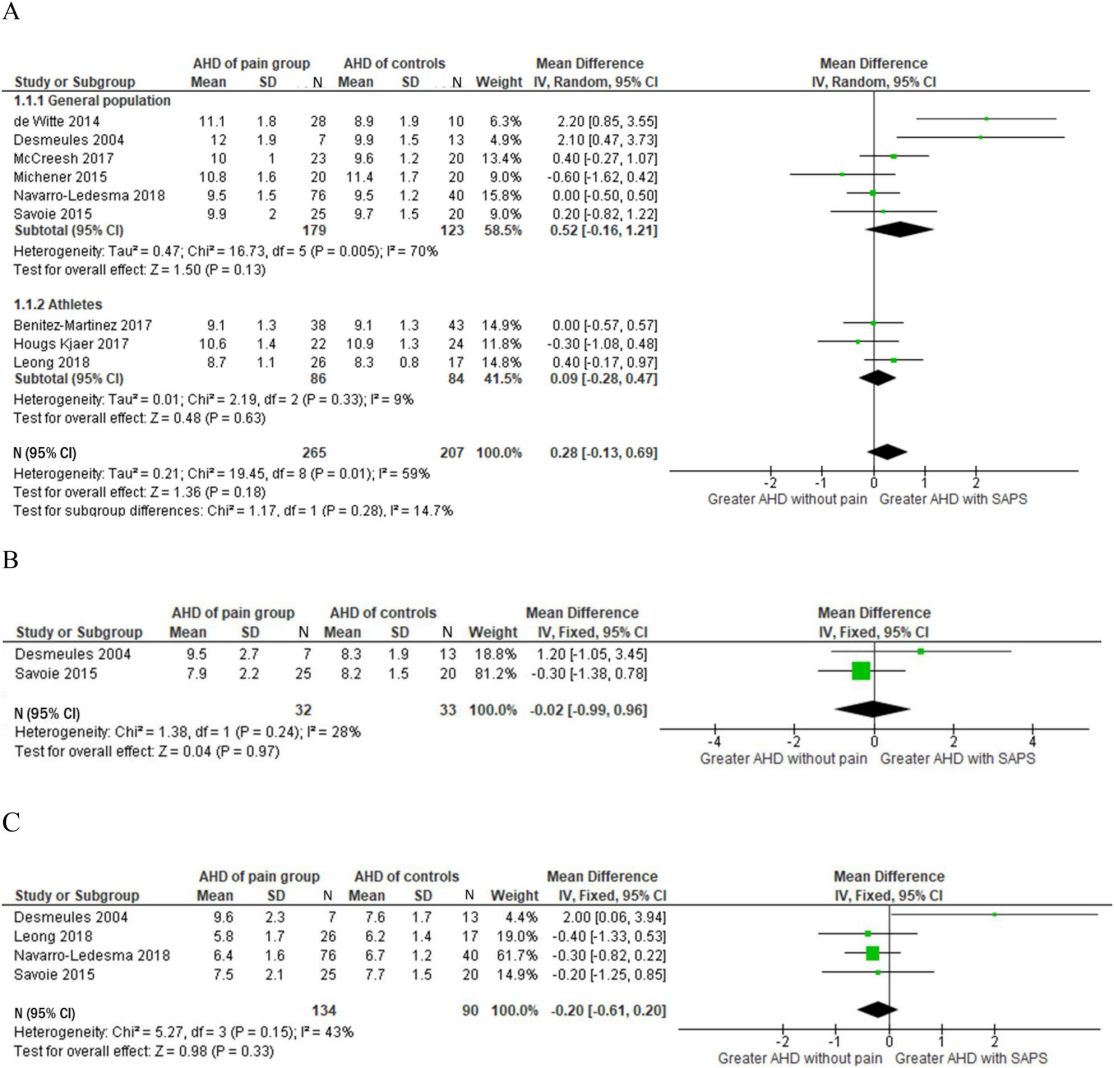
Forest plots comparing AHD (mm) in participants with SAPS and controls with no shoulder pain in varying degrees of shoulder abduction; (**A**) 0°, (**B**) 45°, (**C**) 60°.

There was no between group difference in AHD at 0° of shoulder abduction (MD = 0.28 mm, 95% CI −0.13 to 0.69 mm, I^2^ = 59%) (Fig. 2A). A subgroup analysis of AHD measured at 0° of shoulder abduction in the general population also showed no difference between groups (MD = 0.52 mm, 95% CI −0.16 to 1.21 mm, I^2^ = 70%). Similarly, subgroup analysis performed in the athletic population reported no difference between groups (MD = 0.09 mm, 95% CI −0.28 to 0.47 mm, I^2^ = 9%). As a result of substantial heterogeneity in the overall analysis and in the subgroup analysis of the general population, a sensitivity analysis was performed. Through the visual analysis of the forest plots (Fig. 2A), two studies were identified as studies that may have been responsible for the high heterogeneity^31,32^. The analysis with the exclusion of these studies, substantially reduced the heterogeneity in both analyses (I^2^ = 0%), but the overall meta-analysis continued to demonstrate no difference between groups in the overall population (MD = 0.08 mm, 95% CI −0.17 to 0.33 mm, I^2^ = 0%), as well as general population (MD = 0.06 mm, 95% CI −0.29 to 0.41 mm, I^2^ = 0%).

**Figure 3:**
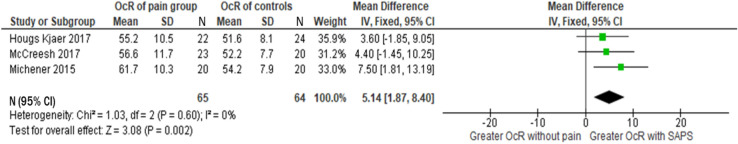
Meta-analysis results comparing occupation ratio percentage in participants with SAPS and controls with no shoulder pain at 0° of shoulder abduction. OcR: Occupation ratio percentage.

There was no difference between groups at 45° of shoulder abduction (MD = −0.02 mm, 95% CI −0.99 to 0.96 mm, I^2^ = 28%) (Fig. 2B), and at 60° of shoulder abduction (MD = −0.20 mm, 95% CI −0.61 to 0.20 mm, I^2^ = 43%) (Fig. 2C). Compared to the control group, occupation ratio was greater in participants with SAPS at 0° of shoulder abduction (MD = 5.14%, 95% CI 1.87 to 8.4%, I^2^ = 0%) (Fig. 3).

### Second aim: correlation between AHD and pain or function

In the three studies that addressed the second aim^54,57,58^, one study reported no correlation (*r* = 0.11, *p* = 0.33) between AHD measured at 0° of shoulder abduction, and pain as measured using the VAS^54^. (Table 2). Another study showed a weak inverse correlation between AHD and SPADI score, at 0° shoulder abduction (*r* = −0.22, *p* < 0.05), meaning that with an increased AHD, there was an improved pain and function, indicated by decreased SPADI score^58^. In the same study, there was no correlation at 60° shoulder abduction (*r* = −0.15, *p* > 0.05)^58^. The results of the third study showed weak and moderate correlation between AHD and the Constant score when AHD was measured at 0° shoulder abduction with both X-ray (*r* = 0.39; *p* = 0.006) and MRI (*r* = 0.41; *p* = 0.004)^57^. This means that with an increased AHD there was an improved pain and function, indicated by increased Constant score.

### Third aim: Changes in subacromial space versus changes in pain/function

Five of the six studies that addressed the third aim^29,59–62^, reported no change in AHD at 0° of shoulder abduction. (Table 5). Similarly, three of the six studies that addressed the third aim^59,61,62^ reported no change in AHD measured at 45° and 60° of shoulder abduction. In comparison, all six studies^29,32,59–62^ reported improvements in pain and disability measured by DASH, SPADI and WORC% scores.

**Table 5:** Summary of third aim.

Author	SAA(°)	Pre AHD (mm)	Post AHD (mm)	*p* value	Change	Outcome measures	Pre pain/disability	Post pain/disabilty	*p* value	Change
Akkaya et al.^59^ (n = 34)	0	11.20 ± 2.30	11.10 ± 2.00	*p* = 0.887	↔	SPADI	62.0 ± 21.5	32.4 ± 18.7	*p* = 0.001	↑
	30	10.50 ± 1.90	10.60 ± 1.90	*p* = 0.257	↔					
						VAS	2.4 ± 2.1	0.9 ± 1.2	*p* = 0.006	↑
	60	10.30 ± 1.90	10.50 ± 2.20	*p* = 0.571	↔					
Belley et al.^60^ (n = 40)	0	10.80 ± 1.70	11.00 ± 1.40	*p* > 0.05	↔	DASH	33.0 ± 13.6	9.5 ± 9.6	*p* < 0.05	↑
	45	8.90 ± 1.40	9.50 ± 1.50	*p* < 0.05	↑^!^					
						WORC%	53.8 ± 18.1%	89.7 ± 12.5%	*p* < 0.05	↑
	60	8.60 ± 1.40	9.30 ± 1.50	*p* < 0.05	↑^!^					
Boudreau et al.^61^ (n = 42)	0	10.8 ± 2.1	11.5 ± 2.7	*p* = 0*.*56	↔	DASH	32.2 ± 15.4	27.8 ± 18.7	*p* > 0.211	↑
	30	10.4 ± 2.3	10.6 ± 2.4	*p* = 0*.*44	↔	WORC%	51.6 ± 18.5%	65.7 ± 25.8%	*p* < 0.001	↑
	60	9.5 ± 2.7	9.8 ± 2.4	*p* = 0*.*75#	↔	VAS	71.1 ± 17.3	56.1 ± 29.6	*p* < 0.001	↑
Dupuis et al.^62^ (n = 43)	0	10 ± 2	10 ± 1	*p* > 0.11	↔	DASH	29.3 ± 12.6	16.3 ± 11.3	*p* < 0.05	↑
	45	8 ± 2	8 ± 2	*p* > 0.11	↔					
						WORC%	55.7 ± 21.0%	80.6 ± 17.1%	*p* < 0.05	↑
	60	7 ± 2	7 ± 2	*p* > 0.11	↔					
Desmeules et al.^32^ (n = 20)	Correlation between max change in AHD (0°–60°) and improvements in WORC % score (*r* = 0.86, *p* = 0.01)									
Savoie et al.^29^ (n = 45)	0	9.9 ± 2.0	10.4 ± 1.7	*p* > 0.05	↔	DASH	Change in DASH score: 17.1 ± 12.4		*p* < 0.001	↑
	45	7.9 ± 2.2	8.8 ± 1.6	*p* < 0.05	↑^!^	WORC%	Change in WORC%score: −30.1 ± 14.0%		*p* < 0.001	↑
	60	7.5 ± 2.1	8.2 ± 1.7	*p* < 0.05	↑^!^					

↔  = no significant difference, # = statistical analysis not reported by authors, calculated using an effect size calculator. ↑ = increased AHD/improved pain & disability. ^!^The increase in acromiohumeral distance (AHD) were found to be smaller than minimal detectable change (MDC = 0.7 mm)^23^ DASH: Disabilities of the Arm Shoulder and Hand questionnaire; SAA: Shoulder abduction angle; SPADI: Shoulder Pain and Disability Index; VAS: Visual Analogue Scale to measure pain; WORC: Western Ontario Rotator Cuff index.

One study^60^ also showed no reduction in occupation ratio at 0° (44.9 ± 7.4% to 44.2 ± 5.2%, *p* > 0.05) of shoulder abduction, but reported reductions in occupation ratio at 45° (54.4 ± 10.3% to 51.6 ± 7.6%, *p* < 0.05) and 60° (56.9 ± 11.0% to 53.1 ± 7.8%, *p* < 0.05) of shoulder abduction.

## Discussion

This systematic review demonstrated no difference in AHD, measured at 0°, 45° and 60° of shoulder abduction, between participants with SAPS and controls with no shoulder pain. This result was consistent across both athletic and the general populations. However, occupation ratio, measured at 0° of shoulder abduction, was marginally greater in participants with SAPS compared to controls with no shoulder pain. This review further demonstrated inconclusive results regarding linear correlations between AHD and patient reported outcome measures of symptoms (Constant score, SPADI, VAS), and regarding associations between improvements in patient reported outcome measures of symptoms (DASH, SPADI, VAS, WORC%) with an increase in AHD over time.

As mentioned, the current review found a marginally greater occupation ratio in participants with SAPS compared to controls with no shoulder pain. This finding suggests that soft tissues in the subacromial space may also be important in SAPS. However, this result has to be interpreted with caution as the mean group difference for occupation ratio was approximately 5%. The minimal detectable change (MDC)^55^ for supraspinatus tendon thickness is 8–10% and for AHD, it is 14.7%^63^. Error for the occupation ratio is therefore likely to be compounded as both MDCs for measurement of AHD and supraspinatus tendon thickness are needed to calculate the occupation ratio, suggesting that the between group difference of 5.14% is most likely to be within measurement error. Further, as there is no established minimal clinical important change (MCID) for occupation ratio, it is unknown whether a 5% difference in occupation ratio is clinically relevant.

The lack of difference in subacromial space between participants with SAPS and pain free controls indicates a reduced likelihood of extrinsic mechanism to SAPS, i.e. pain arising from impingement due to reduced available subacromial space. If SAPS were caused by compression of structures within the subacromial space then a reduced AHD or greater occupation ratio would be expected. However, neither of these were found in this systematic review. This is consistent with a recent systematic review^19^ that summarised the evidence of subacromial decompression surgery with placebo arthroscopy surgery or non-invasive treatments such as exercise, in reducing pain and increasing function in participants with SAPS. This prior systematic review demonstrated that surgical intervention aimed at increasing the subacromial space does not offer additional important benefits when compared with placebo surgery and exercise therapy.

The current systematic review found no consistent pattern of evidence regarding a linear correlation between decreased AHD and improved pain or function. Only one study^54^ compared AHD to pain, only two studies^57,58^ compared AHD to shoulder function and only one study^57^ assessed the shoulder in elevation at 60 degrees. Furthermore, the inconsistent pattern may be explained by the differences in outcome measures (VAS, Constant score, SPADI) and population between the studies. One study^54^ only included an athletic population whereas the participants included in another study^57^ belonged to general population, were of older age, had failed 6 months of conservative management and were awaiting surgery. Additionally, the correlations were generated from studies with relatively low participant numbers and could not be pooled. As a result of these issues (heterogeneity in outcome measures and methodology, populations, and also a possible lack of power), there is limited evidence to determine if AHD is correlated with shoulder pain or function.

This systematic review also found no consistent pattern of whether improvements in patient reported outcome measures are accompanied by increases in AHD over time. The average increase in AHD reported in the included studies^29,59–62^ was 0.28 mm and 0.34 mm respectively, for 0° and 60° of abduction. Both of these values are smaller than the raw MDC value of 0.7 mm^23^ associated with the measurement of AHD and may therefore be ascribed to measurement errors. Despite these small changes in AHD, patient reported outcome measures were improved with management strategies or time in all included studies in the third aim. This supports the notion that improvements in pain or function may be better explained by other associative factors than changes in AHD.

The overall findings of this systematic review suggest that clinicians and researchers need to focus on other biopsychological factors that may be more pertinent to symptoms in SAPS. For example, weakness of the rotator cuff and scapular muscles has been observed in multiple studies in participants with SAPS^64–72^. In addition, systematic reviews have found rotator cuff and scapular muscle strengthening exercises to provide benefits to pain and function in participants with SAPS^18,73^. Thus it would seem likely that musculotendinous dysfunction, such as rotator cuff tendinopathy^74^ is associated with SAPS and is worthy of further research and clinical focus. Other biopsychosocial factors that may be more pertinent include mental and emotional health, as this are associated with self-reported pain and functional measures in those with shoulder pain^75^.

One of the limitations of this review was the relatively small number of studies that investigated occupation ratio. For the second and third aims there were also limited number of studies and high methodological heterogeneity. A further limitation was that only one study^57^ measured subacromial space in shoulder abduction above 60°, and as the painful arc of shoulder abduction movement is observed to be from 60° to 120°^11^, it seems important to examine the associations that contribute to this phenomenon. Although this is described as a limitation, it is recognised that it may be difficult to visualise the subacromial space above 60°, due to imaging limitations below bony structures, in this position.

The strength of this review is that this is the first systematic review with a meta-analysis, synthesising the evidence regarding subacromial space in participants with SAPS and controls with no shoulder pain, with independent article screening and data-extraction. Furthermore, subgroup analysis comparing AHD in the athletic population with and without SAPS was performed. Another strength is that the relationship between subacromial space and symptoms was examined in multiple ways; through a meta-analysis of AHD and occupation ratio, correlations, and associations over time. Moreover, all of the included studies were of moderate to high methodological quality, scoring an average of 75.4% on Downs and Black questionnaire.

It is recommended that future studies focus on exploring the relationship between SAPS and other biopsychosocial factors. Despite the small pooled cohort of 124 participants with SAPS, it was found that the occupation ratio had a small but statistically important relationship to symptom, but presumably within measurement error. However, occupation ratio may be a worthy candidate for further examination in both cross sectional and longitudinal studies.

The findings of this review challenges the previously established theory of pain arising from purely mechanistic etiology for SAPS, as no difference or relations in subacromial space were found between groups, even where improvements in patient reported outcomes were found after interventions. Our findings may be useful in guiding management of patients with SAPS, as the results suggest that management should not focus solely on addressing a potential decrease in subacromial space, but on the importance of other biopsychosocial factors.

## Data Availability

Data for this systematic review is publicly available in the form of published manuscripts, as given in the reference list.

## Appendix: Search Strategy

“Sub-acromial impingement” OR “subacromial impingement syndrome” OR “subacromial impingement” OR “Subacromial pain” OR “subacromial pain syndrome” OR “shoulder impingement syndrome” OR “SAPS” OR “SIS” OR “shoulder impingement” OR “rotator cuff-related shoulder pain” OR “RCRSP” OR “swimmers shoulder” OR “rotator cuff” OR “rotator-cuff” OR “impingement syndrome” OR “superior humeral head migration”.

### AND

“AHD” OR “Subacromial” OR “sub-acromial” OR “Acromiohumeral” OR “acromio-humeral” OR “occupation”.

### AND

“Space” OR “distance” OR “interval” OR “characteristics” OR “ratio” OR “parameters”.
